# How do patient-reported outcome measures affect treatment intensification and patient satisfaction in the management of psoriatic arthritis? A cross sectional study of 503 patients

**DOI:** 10.1093/rheumatology/kead679

**Published:** 2024-01-08

**Authors:** Conor Coyle, Lily Watson, Caroline Whately-Smith, Mel Brooke, Uta Kiltz, Ennio Lubrano, Ruben Queiro, David Trigos, Jan Brandt-Juergens, Ernest Choy, Salvatore D’Angelo, Andrea Delle Sedie, Emmanuelle Dernis, Théo Wirth, Sandrine Guis, Philip Helliwell, Pauline Ho, Axel Hueber, Beatriz Joven, Michaela Koehm, Carlos Montilla Morales, Jon Packham, Jose Antonio Pinto Tasende, Julio Ramírez, Adeline Ruyssen-Witrand, Rossana Scrivo, Sarah Twigg, Martin Welcker, Martin Soubrier, Laure Gossec, Laura C Coates

**Affiliations:** Oxford University, John Radcliffe Hospital, Oxford University Hospitals, Oxford, UK; Department of Cellular and Molecular Medicine, University of Bristol, Bristol, UK; Consultant Biostatistician, Whately-Smith Ltd, Herts, UK; Royal National Hospital for Rheumatic Diseases, Royal United Hospitals, Bath, UK; Ruhr-Universität Bochum, and Rheumazentrum Ruhrgebiet, Bochum, Germany; Academic Rheumatology Unit, Department of Medicine and Health Sciences “Vincenzo Tiberio”, University of Molise, Campobasso, Italy; Rheumatology & ISPA Translational Immunology Division, Faculty of Medicine, Rheumatology Service & the Principality of Asturias Institute for Health Research (ISPA), Universidad de Oviedo, Oviedo, Spain; Acción Psoriasis, Barcelona, Spain; Rheumatologische Schwerpunktpraxis, Berlin, Germany; CREATE Centre, Division of Infection and Immunity, Cardiff University, Cardiff, UK; Rheumatology Department of Lucania, San Carlo Hospital, Potenza, Italy; Rheumatology Unit, Azienda Ospedaliero Universitaria Pisana, Pisa, Italy; Rheumatology Department, Centre Hospitalier du Mans, Le Mans, France; Rheumatology Department, INSERM UMRs1097 Autoimmune Arthritis, Aix Marseille University, Marseille, France; Rheumatology Department, Aix Marseille University, Arthrites Autoimmunes, Marseille, France; Leeds Institute of Rheumatic and Musculoskeletal Medicine, University of Leeds, Leeds, UK; The Kellgren Centre for Rheumatology, Manchester University NHS Foundation Trust, Manchester, UK; Division of Rheumatology, Klinikum Nürnberg, Paracelsus Medical University, Nürnberg, Germany; Rheumatology Department, Hospital Universitario 12 Octubre, Madrid, Spain; Universidad Complutense de Madrid, Avda de Córdoba sin, Madrid, Spain; Rheumatology, Universitiy Hospital Frankfurt, Fraunhofer-Institute for Translational Medicine and Cluster of Excellence Immune-Mediated Diseases CIMD, Frankfurt am Main, Germany; Rheumatology Department, Hospital Universitario Salamanca, Salamanca, Spain; Academic Unit of Population and Lifespan Sciences, University of Nottingham, Nottingham, UK; Rheumatology Department, INIBIC. CHU A Coruña, A Coruña, Spain; Arthritis Unit, Rheumatology Department, Hospital Clinic, Barcelona, Spain; Rheumatology Center, Toulouse University Hospital, CIC 1436 Inserm, Rheumatology, Tolouse, France; Paul Sabatier University, Toulouse III, Toulouse, France; Rheumatology Unit, Department of Clinical Internal, Anesthesiological and Cardiovascular Sciences, Sapienza University of Rome, Rome, Italy; Rheumatology, Bradford Teaching Hospitals NHS Foundation Trust, Bradford, UK; MVZ für Rheumatologie Dr M. Welcker GmbH, Planegg, Germany; Rheumatology Department, Gabriel-Montpied Teaching Hospital of Clermont-Ferrand, Clermont-Ferrand, France; Rheumatology Department, Sorbonne Université, INSERM, Institut Pierre Louis d’Epidémiologie et de Santé Publique, Paris, FR. & AP-HP, Pitié-Salpêtrière Hospital, Paris, France; Nuffield Department of Orthopaedics, Rheumatology and Musculoskeletal Sciences, University of Oxford, Oxford, UK

**Keywords:** psoriatic arthritis, PsA, quality of life, PsAID, PsAID-12, ASSIST, HAQ, EQ-5D-5L, patient-reported outcomes

## Abstract

**Objectives:**

The AsseSSing Impact in pSoriatic Treatment (ASSIST) study investigated prescribing in routine PsA care and whether the patient-reported outcome—PsA Impact of Disease questionnaire (PsAID-12)—impacted treatment. This study also assessed a range of patient and clinician factors and their relationship to PsAID-12 scoring and treatment modification.

**Methods:**

Patients with PsA were selected across the UK and Europe between July 2021 and March 2022. Patients completed the PsAID questionnaire and the results were shared with their physician. Patient characteristics, disease activity, current treatment methods, treatment strategies, medication changes and patient satisfaction scores were recorded.

**Results:**

A total of 503 patients were recruited. Some 36.2% had changes made to treatment, and 88.8% of these had treatment escalation. Overall, the mean PsAID-12 score was higher for patients with treatment escalation; increase in PSAID-12 score is associated with increased odds of treatment escalation (odds ratio 1.58; *P* < 0.0001). However, most clinicians reported that PsAID-12 did not impact their decision to escalate treatment, instead supporting treatment reduction decisions. Physician’s assessment of disease activity had the most statistically significant effect on likelihood of treatment escalation (odds ratio 2.68, per 1-point score increase). Escalation was more likely in patients not treated with biologic therapies. Additional factors associated with treatment escalation included: patient characteristics, physician characteristics, disease activity and disease impact.

**Conclusion:**

This study highlights multiple factors impacting treatment decision-making for individuals with PsA. PsAID-12 scoring correlates with multiple measures of disease severity and odds of treatment escalation. However, most clinicians reported that the PsAID-12 did not influence treatment escalation decisions. Psoriatic Arthritis Impact of Disease (PsAID) scoring could be used to increase confidence in treatment de-escalation.

Rheumatology key messages

- This study highlights multiple factors on decision-making when reviewing treatments for individuals with PsA.
- The heterogeneity of clinical phenotype, with increasing number of effective therapies, necessitates collaborative treatment decision-making.

## Introduction

PsA is a chronic musculoskeletal inflammatory disease [1]. As a result of the diversity of clinical presentation and treatment responsiveness there is often need for personalization of the therapeutic approach. Currently little is known about the factors underpinning treatment choices in routine practice [2, 3].

Patient-reported outcome (PRO) measures have been developed to measure disease activity, both guiding treatment decisions in clinical standard and standardizing outcomes in clinical research [4]. The PsA Impact of Disease questionnaire (PsAID) is a disease-specific PRO co-designed by clinicians and patients to measure the overall impact of psoriatic disease from the patient perspective and also put forward in OMERACT and Group for Research and Assessment of Psoriasis and Psoriatic Arthritis (GRAPPA) meetings [5–7]. There are two versions of the PRO: a 9-item questionnaire for use in clinical trials and a longer 12-item questionnaire with simplified scoring for clinical practice (PsAID-12) [2]. The PsAID-12 was designed for use in clinical practice to monitor patients and identify areas that might require intervention in ongoing clinical management. It has been validated in a number of observational studies and interventional trials [5, 8–11]. The MEdir Resultados. Consenso de Evaluación en Salud para artritis psoriásica (MERECES) study proposed PsAID as a standard tool for evaluating the impact of disease and also as an essential instrument in making therapeutic decisions in PsA [12]. However, there are limited data on its use in routine practice.

The purpose of the AsseSSing Impact in pSoriatic Treatment (ASSIST) study was to investigate the prescribing practice for PsA in routine care and whether the use of the PRO PsAID-12, impacted treatment decisions in the post-COVID-19 era.

To understand more about the consultations of patients with PsA and factors that underpin decisions to change treatment, we also recorded measures of satisfaction in consultation and measure of shared decision-making in practice. By comparing treatment data between countries, we can understand more about factors influencing the treatments patients receive and patient outcomes, and establish international benchmarks in practice.

## Methods

The ASSIST study was a cross-sectional analysis of adult patients aged 18 years and older, attending a face-to-face rheumatology appointment, with a clinical diagnosis of PsA made previously by a rheumatologist (meeting the Classification of Psoriatic Arthritis criteria) [11]. Patients were selected by systematic sampling from 24 centres across five countries (UK, France, Germany, Italy and Spain) between July 2021 and March 2022. Ethical approval was specifically gained for this research study via London—Camden & Kings Cross Research Ethics Committee research: Ethics reference: 20/PR/0587, and has been listed via the IRAS platform (IRAS ID: 287039).

### Patients

Patients were aged 18 years and older attending a face-to-face appointment, with a known diagnosis of PsA made by a rheumatologist. Patients were excluded from the study if they had a new diagnosis of PsA at the current clinic visit; were not comfortable completing an app-based questionnaire or paper case-report form; or were unable to speak/read the local language. Given our aim to analyse factors underpinning treatment decisions, a target sample size of 100 patients per country was chosen based on data that 32% of patients undergo a treatment change at a clinic appointment [12].

Each centre aimed to recruit the same number of patients. Patients were selected using systematic sampling with random starting numbers generated for each site. Participants gave written informed consent.

The primary objective was to assess the influence of the PsAID-12 score on likelihood of treatment escalation. Therefore, the PsAID questionnaire was completed by the patient prior to the appointment and the scores shared with the treating physician in their standard appointment. Patients were treated in their routine clinical practice. Patient and disease characteristics, current treatment methods and decisions on treatment strategies (medications unchanged, switched, added or reduced) were recorded.

This study was developed to look at different aspects of the disease and the associations between these and treatment change. Patient and disease characteristics were recorded, including: patient demographics, PsA duration, prior and current treatment, number of comorbidities (according to the functional comorbidity index [13]) and disease activity. Composite scores have previously been shown to be associated with treatment change [12], however they were not used in this study to enable clarity in looking at separate (and different) aspects of the disease in greater detail and the association of these with treatment change.

Disease activity measures included:

- a clinical assessment including clinical history which included duration of disease and prior and current treatment;
- tender and swollen joint count (the inclusion of axial spine disease within this pragmatic study was at the discretion of the acting clinician and their assessment of active disease within their routine clinical practice; no direct data were recorded on this);
- dactylitis count;
- body surface area of psoriasis;
- physician-rated overall assessment of disease activity score;
- Widespread Pain Index and Severity Scale for FM; and
- Leeds Enthesitis Index [13–15].

Participants completed PROs prior to their clinic appointment, including:

- the PsAID-12 questionnaire via the GRAPPA app on a tablet (scored from 0 to 10, with 10 reflecting worst possible health);
- the numerical rating scale for disease activity and pain;
- the HAQ; and
- the EuroQoL 5-Dimension 5-Level (EQ-5D-5L) [6, 16].

PsAID-12 scores were shared with the treating physician during the appointment. Current treatment methods and treatment decisions (treatment unchanged, escalated or reduced) were recorded. Escalation was defined as one or more of the following: increase in current medication dose; increase in medication frequency; change in route of administration; addition of a new medication; or switch to a new medication.

Comorbidities were summarized for each patient using the functional comorbidity index (FCI) [13]. A total score was obtained by counting the number of conditions present (range of 0–18). If at least one condition was not classified as present or absent, then the total score was set to missing. Conditions included in the scoring criteria included: arthritis (RA and OA); osteoporosis; asthma; chronic obstructive pulmonary disease (including acquired respiratory distress syndrome and emphysema); angina; congestive heart failure (or heart disease); heart attack (myocardial infarction); neurological disease (Parkinson’s or multiple sclerosis); stroke or transient ischaemic attack; peripheral vascular disease; diabetes (type 1 or type 2); upper gastrointestinal disease (ulcer, hernia, reflux); depression; anxiety or panic disorders; visual impairment (cataracts or glaucoma); hearing impairment; degenerative disc disease (back disease, spinal stenosis or severe chronic back pain) obesity or BMI over 30 kg/m^2^. (The number of comorbidities was generally low, median of 1, with no patient having >11.)

Participants completed PROs including the PsAID-12 questionnaire (administered using the GRAPPA app on a tablet), numerical rating scale for disease activity and pain, the HAQ and EQ-5D-5L [16].

After visits, patients independently completed the following questionnaires:

- the CollaboRATE questionnaire, which examines the patients perception of shared-decision-making (scored from 0 to 9) [7, 12, 17]; and
- the Perceived Efficacy in Patient Physician Interactions (PEPPI) tool, which assesses the patients’ view on their confidence in the patient–doctor interaction (scored from 5 to 25) [18, 19]. Clinicians were asked to rate six possible factors influencing their treatment choice in each case: joint/entheseal activity, skin disease activity, PROs, tolerance of current medication and adherence to current medication.

These two questionnaires were completed by the patients independently and the completed questionnaires were not seen by clinic staff to avoid any influence being exerted on the patients.

At the end of the study, each participating physician was asked to provide their views on the PsAID-12 instrument. Brief details of the participating centres were collected, including the size of the PsA population at the site, as well as demographic details of the physicians.

Our primary outcome variable was escalation of PsA treatment by the clinician. Escalation was defined as one or more of the following treatment decisions being made at the study visit: increase in dose of current medication; increase in frequency of dose administration; change in route of administration; initiation of a new medication; or initiation of a new medication as a switch from existing DMARD therapy.

Secondary outcome variables included: PsAID-12 score; CollaboRATE satisfaction with consultation; and PEPPI [1, 11, 20]. The PEPPI tool was used to assess the patients’ view on their confidence in the patient–doctor interaction.

This study also aimed to evaluate the impact of reviewing the PsAID-12 score on the decision to change treatment; assess the effects of other factors that influence the likelihood of treatment escalation; determine which factors physicians feel influence treatment decisions in routine practice; and evaluate patient satisfaction and perceived patient efficacy in the consultation and examine how this links to PsAID-12 score and change in treatment. We also looked to explore physicians’ views on the use and value of the PsAID-12 tool.

Each centre aimed to recruit the same number of patients aiming at 100 patients per country. Patients were selected using systematic sampling with random starting numbers generated for each site.

### Statistical analysis

Statistical analysis was completed on SAS^®^ Version 9.4 [21]. There was no imputation of missing data. The initial sample size calculation was based on the need to estimate the percentage of patients for whom treatment was modified, with a stated degree of precision. This was defined as a 95% CI for the percentage with width 10 percentage points. This is based on data from the GRACE (GRAPPA Composite Exercise) study which recruited 503 patients worldwide and found that 32% underwent a treatment change, the majority being escalation for active disease [22]. For a percentage of 30% (i.e. 30% of patients requiring treatment change), a study of 333 patients would have 80% power to estimate a percentage of 30% requiring change with a CI of ±5%.

The overall probability of treatment being escalated predicted by the mean PsAID score, adjusted for clinic, was estimated along with associated 95% CI. The effect of the total PsAID score on the probability of modifying treatment, adjusting for clinic, was expressed as an odds ratio (OR) for unit increases in PsAID score with associated 95% CI. To assess the effect of the PsAID total score on treatment escalation, the total score was added to the basic logistic regression model as an independent continuous variable. The same sampling weights and variance estimation method were used as described above for the basic model. The effect of PsAID was then assessed by comparing the deviance for the two models.

## Results

There were 503 patients recruited from 24 centres (49.1% female; mean age 53 years; median patient age 55 years) (Table 1). Mean disease duration was 10.8 (s.d. 9.28) years. The most common PsA subtype was peripheral arthritis in all countries (83.7%). The mean physicians’ assessment of disease activity across countries was 3.0 (range 0–9), indicating that disease severity was generally mild (Table 1). The level of disability was also low, with mean scores of 0.6 on the HAQ score, a median tender joint count of 2 and median swollen joint count of 0. Overall, the mean total PsAID score was 3.6. Notably, both physician- and patient-reported outcomes in the UK indicated higher levels of disease activity and disease impact than other European countries (Table 1).

**Table 1.** Patient characteristics

	France (*n* = 100)	Germany (*n* = 101)	Italy (*n* = 84)	Spain (*n* = 111)	UK (*n* = 107)	All (*n* = 503)
Age (years)						
Mean	54.9	55.3	54.3	53.8	51.6	53.9
Median	55.0	56.0	55.0	56.0	51.0	55.0
s.d.	12.44	12.12	11.74	11.47	13.56	12.33
Min–Max	29–83	22–81	21–81	18–79	28–80	18–83
Sex, *n* (%)						
Female	47 (47.0)	58 (57.4)	29 (34.5)	54 (48.6)	59 (55.1)	247 (49.1)
Male	53 (53.0)	43 (42.6)	55 (65.5)	57 (51.4)	48 (44.9)	256 (50.9)
No. of comorbidities (FCI)						
Mean	1.5	1.4	1.2	1.3	1.5	1.4
Median	1.0	1.0	1.0	1.0	1.0	1.0
s.d.	1.61	1.51	1.10	1.64	1.70	1.54
Min–Max	0–7	0–7	0–4	0–7	0–11	0–11
No. of comorbidities (FCI category), *n* (%)						
0	34 (34.0)	33 (32.7)	25 (29.8)	47 (42.3)	33 (30.8)	172 (34.2)
1	24 (24.0)	26 (25.7)	34 (40.5)	25 (22.5)	25 (23.4)	134 (26.6)
2	16 (16.0)	22 (21.8)	12 (14.3)	19 (17.1)	19 (17.8)	88 (17.5)
3	12 (12.0)	7 (6.9)	10 (11.9)	5 (4.5)	15 (14.0)	49 (9.7)
4	7 (7.0)	3 (3.0)	3 (3.6)	4 (3.6)	4 (3.7)	21 (4.2)
5	2 (2.0)	4 (4.0)	0 (0.0)	3 (2.7)	0 (0.0)	9 (1.8)
6	2 (2.0)	1 (1.0)	0 (0.0)	2 (1.8)	1 (0.9)	6 (1.2)
7	1 (1.0)	1 (1.0)	0 (0.0)	2 (1.8)	1 (0.9)	5 (1.0)
11	0 (0.0)	0 (0.0)	0 (0.0)	0 (0.0)	1 (0.9)	1 (0.2)
Duration of disease (years)						
Mean	12.8	9.0	11.8	11.0	9.7	10.8
Median	10.0	7.0	8.5	9.0	7.0	8.0
s.d.	9.64	8.45	11.15	8.67	8.34	9.28
Min–Max	1–63	1–41	1–56	1–50	0–36	0–63

FCI: Functional Comorbidity Index.

Current prescribing practices are shown in Table 2. Notably, a higher percentage of UK patients are managed with conventional synthetic DMARDS than mainland Europe (66.4% *vs* 44.9%), whereas use of biologics is more frequent in mainland Europe than the UK (68.1% *vs* 36.4%). Overall, treatment was changed for 182 patients (36.2%), with an increase in treatment being the most common type of change in this group (160 patients, 88.8%) (Table 3). The treatment increase consisted of medication addition (14.1%), medication switch (10.7%), or an increase in dose, frequency or change in route from oral to s.c. MTX (9.3%). Notably, treatment escalation was more common in the UK than Europe, commonly being a treatment escalation. This may reflect the higher level of physician- and patient-reported disease activity, the predominance of conventional synthetic DMARD use or the younger patient demographic in the UK, as treatment escalation is more likely earlier in the disease course.

**Table 2.** Current PsA treatment

	France (*n* = 100)	Germany (*n* = 101)	Italy (*n* = 84)	Spain (*n* = 111)	UK (*n* = 107)	All (*n* = 503)
Conventional synthetic DMARDs, *n* (%)						
Any DMARDS	52 (52.0)	45 (44.6)	27 (32.1)	54 (48.6)	71 (66.4)	249 (49.5)
MTX	43 (43.0)	38 (37.6)	23 (27.4)	40 (36.0)	50 (46.7)	194 (38.6)
LEF	3 (3.0)	3 (3.0)	1 (1.2)	6 (5.4)	4 (3.7)	17 (3.4)
SSZ	0 (0.0)	0 (0.0)	4 (4.8)	6 (5.4)	19 (17.8)	29 (5.8)
Other	4 (4.0)	3 (3.0)	2 (2.4)	2 (1.8)	5 (4.7)	16 (3.2)
Biologics, *n* (%)						
Any biologics	63 (63.0)	69 (68.3)	62 (73.8)	69 (62.2)	39 (36.4)	302 (60.0)
Etanercept	7 (7.0)	10 (9.9)	11 (13.1)	6 (5.4)	7 (6.5)	41 (8.2)
Adalimumab	9 (9.0)	16 (15.8)	12 (14.3)	20 (18.0)	13 (12.1)	70 (13.9)
Infliximab	10 (10.0)	0 (0.0)	1 (1.2)	7 (6.3)	0 (0.0)	18 (3.6)
Golimumab	5 (5.0)	5 (5.0)	7 (8.3)	3 (2.7)	3 (2.8)	23 (4.6)
Certolizumab	6 (6.0)	1 (1.0)	2 (2.4)	1 (0.9)	3 (2.8)	13 (2.6)
Secukinumab	7 (7.0)	16 (15.8)	11 (13.1)	11 (9.9)	7 (6.5)	52 (10.3)
Ixekizumab	2 (2.0)	7 (6.9)	12 (14.3)	9 (8.1)	0 (0.0)	30 (6.0)
Ustekinumab	10 (10.0)	5 (5.0)	4 (4.8)	7 (6.3)	2 (1.9)	28 (5.6)
Other	5 (5.0)	8 (7.9)	1 (1.2)	5 (4.5)	3 (2.8)	22 (4.4)
Oral glucocorticoids, *n* (%)						
Any glucocorticoids	2 (2.0)	10 (9.9)	9 (10.7)	10 (9.0)	6 (5.6)	37 (7.4)
Prednisolone	1 (1.0)	10 (9.9)	5 (6.0)	2 (1.8)	5 (4.7)	23 (4.6)
Other	1 (1.0)	0 (0.0)	3 (3.6)	7 (6.3)	1 (0.9)	12 (2.4)

Percentages calculated using the total number of patients in each country or overall.

Patients may be on more than one treatment so percentages will not sum to 100.

**Table 3.** Treatment decision made at visit

	France (*n* = 100)	Germany (*n* = 101)	Italy (*n* = 84)	Spain (*n* = 111)	UK (*n* = 107)	All (*n* = 503)
Change in PsA treatment, *n* (%)						
No	70 (70.0)	67 (66.3)	60 (71.4)	72 (64.9)	52 (48.6)	321 (63.8)
Yes	30 (30.0)	34 (33.7)	24 (28.6)	39 (35.1)	55 (51.4)	182 (36.2)
Increase	28 (28.0)	26 (25.7)	20 (23.8)	35 (31.5)	51 (47.7)	160 (31.8)
Decrease	2 (2.0)	8 (7.9)	4 (4.8)	4 (3.6)	4 (3.7)	22 (4.4)
Increase^a^, *n* (%)						
Dose	8 (8.0)	4 (4.0)	3 (3.6)	7 (6.3)	8 (7.5)	30 (6.0)
Frequency	3 (3.0)	4 (4.0)	0 (0.0)	3 (2.7)	1 (0.9)	11 (2.2)
Route change	1 (1.0)	0 (0.0)	0 (0.0)	4 (3.6)	1 (0.9)	6 (1.2)
Additional medication	9 (9.0)	12 (11.9)	6 (7.1)	16 (14.4)	28 (26.2)	71 (14.1)
Replacement medication	8 (8.0)	9 (8.9)	13 (15.5)	9 (8.1)	15 (14.0)	54 (10.7)
Decrease^a^, *n* (%)						
Dose	0 (0.0)	5 (5.0)	0 (0.0)	1 (0.9)	0 (0.0)	6 (1.2)
Frequency	1 (1.0)	1 (1.0)	2 (2.4)	0 (0.0)	0 (0.0)	4 (0.8)
Route change	0 (0.0)	0 (0.0)	0 (0.0)	0 (0.0)	0 (0.0)	0 (0.0)
Stop medication	1 (1.0)	2 (2.0)	2 (2.4)	3 (2.7)	4 (3.7)	12 (2.4)

Percentages calculated using the total number of patients in each country or overall.

^a^There can be more than one reason for type of change so percentages will not add up to 100.

**Figure 1.**
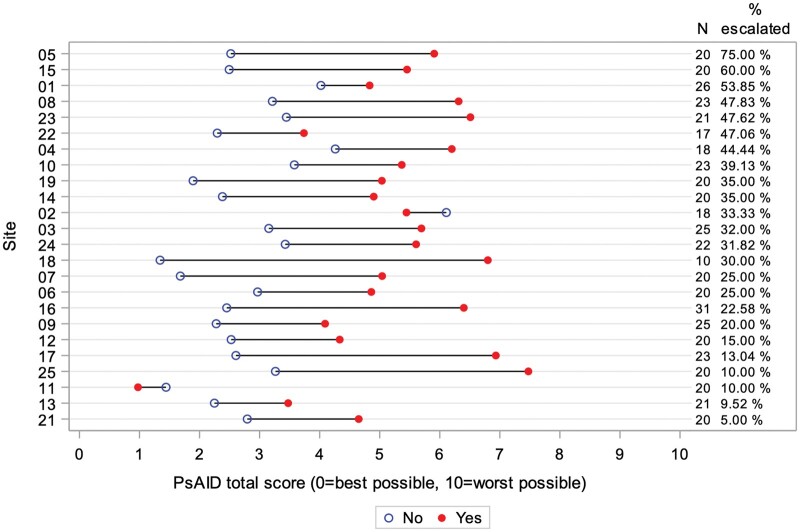
Mean PsAID score by treatment escalation: graph demonstrating decision of treatment escalation in relation to PsAID score, by treatment site. PsAID: PsA Impact of Disease questionnaire

**Figure 2.**
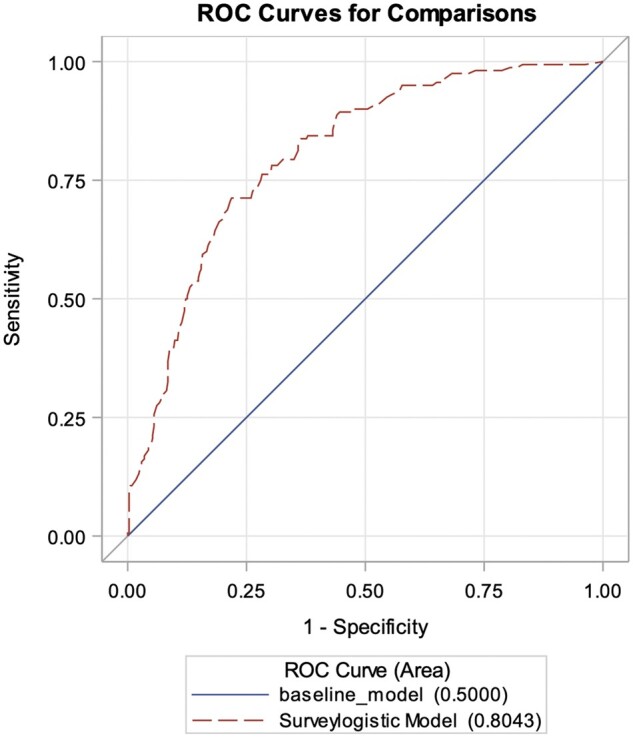
ROC curves for comparisons: ROC curve as a graphical demonstration of the usefulness of PsAID as a predictor for treatment escalation. ROC: receiver operating characteristic; PsAID: PsA Impact of Disease questionnaire

When examining the relationship between PsAID-12 score and treatment escalation, we found that the mean PsAID-12 score for patients with treatment escalation was higher than that for those without escalation in 22/24 sites (Fig. 1). The PsAID-12 score was associated with the odds of treatment escalation (OR 1.58; *P* < 0.0001), reflecting that the estimated odds of treatment escalation increased by 58% with every 1-point increase in the score. A receiver operating characteristic curve (Fig. 2) demonstrates the value of the PsAID score as a predictor of treatment escalation.

Overall, the mean total PsAID score was 3.6. The mean physicians’ assessment of disease activity was 3.0 (range 0–9) for all countries, indicating that disease severity was generally mild (Table 4). The level of disability was low, with mean scores of 0.6 on the HAQ score. However, both physician- and patient-reported outcomes showed higher levels of disease activity and impact in patients recruited in the UK (Table 4). Across the cohort, 62.2% of patients had at least one comorbidity (Table 2).

**Table 4.** Current PsAID status with patient reported outcome scores

	France (*n* = 100)	Germany (*n* = 101)	Italy (*n* = 84 )	Spain (*n* = 111)	UK (*n* = 107)	All (*n* = 503)
Body surface area affected, *n* (%)						
Clear	37 (37.0)	39 (38.6)	28 (33.3)	37 (33.3)	34 (31.8)	175 (34.8)
<=3%	54 (54.0)	60 (59.4)	39 (46.4)	71 (64.0)	63 (58.9)	287 (57.1)
3.1-10%	4 (4.0)	2 (2.0)	14 (16.7)	2 (1.8)	9 (8.4)	31 (6.2)
10.1-15%	2 (2.0)	0 (0.0)	3 (3.6)	0 (0.0)	0 (0.0)	5 (1.0)
>15%	3 (3.0)	0 (0.0)	0 (0.0)	1 (0.9)	1 (0.9)	5 (1.0)
Leeds Enthesitis (Score), *n* (%)						
0	70 (70.0)	86 (85.1)	54 (64.3)	81 (73.0)	68 (63.6)	359 (71.4)
1	5 (5.0)	5 (5.0)	10 (11.9)	7 (6.3)	12 (11.2)	39 (7.8)
2	15 (15.0)	6 (5.9)	7 (8.3)	10 (9.0)	12 (11.2)	50 (9.9)
3	1 (1.0)	0 (0.0)	3 (3.6)	3 (2.7)	3 (2.8)	10 (2.0)
4	5 (5.0)	2 (2.0)	8 (9.5)	2 (1.8)	3 (2.8)	20 (4.0)
5	0 (0.0)	0 (0.0)	0 (0.0)	1 (0.9)	1 (0.9)	2 (0.4)
6	2 (2.0)	0 (0.0)	2 (2.4)	1 (0.9)	3 (2.8)	8 (1.6)
Tender joint count						
Mean	3.5	2.7	3.1	2.6	6.7	3.8
Median	1.0	0.0	2.0	1.0	3.0	2.0
s.d.	5.54	5.32	3.42	3.33	11.09	6.67
Min−Max	0−30	0−28	0−13	0−20	0−66	0−66
Swollen joint count						
Mean	0.7	0.5	0.8	1.3	2.4	1.2
Median	0.0	0.0	0.0	0.0	1.0	0.0
s.d.	1.93	1.47	1.28	2.20	3.29	2.30
Dactylitis count						
Mean	0.1	0.1	0.2	0.1	0.3	0.1
Median	0.0	0.0	0.0	0.0	0.0	0.0
s.d.	0.28	0.48	0.53	0.39	1.05	0.62
Min−Max	0−2	0−3	0−3	0−3	0−8	0−8
Physician’s overall assessment of disease activity						
Mean	2.7	2.6	2.8	3.1	3.7	3.0
Median	2.0	2.0	2.0	3.0	4.0	3.0
s.d.	2.06	2.08	2.25	2.22	2.33	2.22
Min−Max	0−8	0−9	0−8	0−8	0−8	0−9
PsAID (total, calculated from scores)^a^						
Mean	3.76	2.80	3.17	3.53	4.81	3.63
Median	3.65	2.05	2.58	3.25	5.30	3.50
s.d.	2.420	2.220	2.510	2.206	2.560	2.469
Min−Max	0.00−7.80	0.00−8.40	0.00−9.25	0.10−9.35	0.00−9.80	0.00−9.80
PsAID (total, from GRAPPA app)^a^						
Mean	3.68	2.66	3.02	3.33	4.60	3.48
Median	3.65	2.00	2.50	3.05	5.13	3.33
s.d.	2.378	2.149	2.490	2.138	2.555	2.426
Min−Max	0.00−7.92	0.00−8.00	0.00−9.20	0.08−9.35	0.00−9.75	0.00−9.75
HAQ (total, alternative calculation)^b^						
Mean	0.615	0.474	0.501	0.620	0.936	0.636
Median	0.500	0.250	0.250	0.500	0.875	0.500
s.d.	0.603	0.529	0.545	0.571	0.756	0.629
Min−Max	0.000−2.250	0.000−2.125	0.000−2.250	0.000−2.875	0.000−2.625	0.000−2.875
EQ-VAS^c^						
Mean	63.0	63.8	64.3	66.6	59.2	63.4
Median	60.0	70.0	70.0	70.0	60.0	65.0
s.d.	20.83	26.13	23.07	18.64	21.04	22.03
Min−Max	6−100	5−100	5−100	10−100	20−98	5−100
No data	0	1	1	1	2	5

a PsAID: 0 to 10, where 0 = Best possible score, 10 = Worst possible score

b HAQ alternative disability index: 0 to 3, where 0 = Best possible score, 3 = Worst possible score. Total derived from worst scores in each category

c EQ-5D, VAS for current health: 0 to 100, where 0 = Worst possible score, 100 = Best possible score

Generally, levels of disease activity were low, with a median tender joint count of 2 and swollen joint count of 0. The overall percentage of patients with predominantly enthesitis was 4.8%, with the highest percentages seen in Italy (7.1%) and France (7.0%). The dactylitis scores were similarly low, with most patients in all countries scoring 0. In keeping with a rheumatology clinic population, the majority of patients (91.9%) with a body surface area of psoriasis <3% (Table 4).

**Figure 3.**
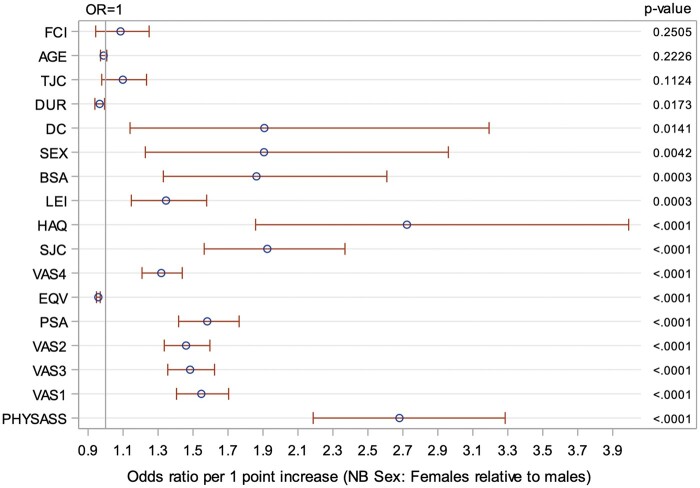
Effect of each variable on the odds of treatment escalation: univariate analysis showing effect of each variable on the odds of treatment escalation. OR: odds ratio; FCI: Functional Comorbidity Index; AGE: age (years); TJC: tender joint count; DUR: disease duration; DC: dactylitis count; SEX: sex; BSA: body surface area psoriasis; LEI: Leeds Enthesitis Index; HAQ: Health Assessment Questionnaire; SJC: swollen joint count; VAS4: patient reported skin psoriasis activity; EQV: EQ-5D-5L VAS score; PSA: PsA Impact of Disease questionnaire; VAS2: patient-reported overall assessment of disease activity; VAS3: patient-reported joint disease severity; VAS1: patient-reported pain score; PHYSASS: physicians assessment of disease activity

The physician’s assessment of disease activity had the most statistically significant effect on the likelihood of treatment escalation, with an OR of 2.68 for each 1-point increase in score. A high level of correlation was found between variables, including physician’s global assessment of disease and the patient-reported PsAID-12 score (correlation of 0.64). Using univariate regression, we identified other factors associated with treatment escalation, including patient characteristics, physician characteristics, disease activity and disease impact (Fig. 3). Treatment escalation was also more likely in patients who were not already treated with biologic therapies. Only age, tender joint count and comorbidity index were not significantly associated with treatment escalation.

Therefore, a multiple logistic regression model was run with a reduced set of potential factors. When all individually significant factors were included, only five factors were significant in multivariable analysis: physician’s assessment, disease duration, non-biological treatment, swollen joint count and EQ Visual Analogue Scale (EQ-VAS). The inclusion of the PsAID-12 score in this model did not materially affect the results.

Clinicians were asked to rate six possible factors influencing their treatment choice in each case: joint/entheseal activity, skin disease activity, PROs, tolerance of current medication and adherence to current medication. Assessment of joint and entheseal disease activity was perceived to have the highest impact on treatment decisions, with markers of systemic inflammation (CRP) being the lowest. In most cases, the clinicians reported that the PsAID score did not significantly influence the decision on treatment escalation beyond these other factors. Where there was an impact on treatment decisions, a review of the PsAID scores was more likely to lead to a decrease in treatment rather than an increase.

The mean CollaboRATE score was 7.96 (maximum possible score 9), indicating a high degree of satisfaction overall, with 52.9% of patients giving the maximum score for satisfaction with their consultation. Generally, PEPPI patient confidence scores were also high with a mean score of 21.4 (maximum possible score 25). Similar mean scores for CollaboRATE and PEPPI were seen in those who did and did not have a treatment escalation. However, in patients with low CollaboRATE scores, treatment escalation only occurred in those with high PsAID scores, whereas in those with high CollaboRATE scores, even patients with low PsAID scores underwent treatment escalation.

## Discussion

To date, the influence of various patient and clinician factors on treatment decisions for PsA in real-world practice has not been examined. The purpose of the ASSIST study was to investigate the prescribing practice for PsA in routine care and whether the use of the PRO PsAID-12 impacted treatment decisions in the post-COVID-19 era. The heterogeneity of clinical phenotype and treatment responsiveness in the PsA cohort, alongside the increasing number of effective therapies necessitates collaborative and personalized treatment decision-making.

In this large, multicentre international analysis, we examine treatment decisions in over 500 participants in routine practice, with a particular focus on the role of the PRO PsAID-12. Generalizability was enhanced by including multiple centres across different countries. Nevertheless, all participants were recruited from specialist PsA clinics and disease activity was generally low, which may differ from other rheumatology clinics. It is likely that results may be different in those with more significant skin disease, although this population does seem to reflect most rheumatology clinic populations [22, 23].

Overall, we found high rates of treatment escalation. One explanation for this is the expansion of treatment options and increasing focus on treat-to-target approaches in recent times. We demonstrate that many aspects of an individual case are considered during treatment decision-making. The single factor most associated with treatment change was physician’s assessment of disease activity, but swollen joint count, previous medications, disease duration and EQ-VAS were also associated with treatment escalation in multivariable analysis. Clinicians reported that joint counts and assessment of enthesitis were the most common drivers of treatment decisions.

We aimed to examine the influence of PsAID-12 score on decision-making. PsAID has been shown to enable prediction of disease flares in new-onset PsA and prediction of achieving treatment objectives, such as the minimal disease activity response [11, 12]. We found that PsAID score correlates with multiple measures of disease severity and there was a significant association between PsAID-12 scores and the odds of treatment escalation. Patients with a higher PsAID-12 score were more likely to have had treatment escalation, however a majority of physicians reported that PsAID-12 had little impact on their clinical decision to escalate treatment.

Most physicians reported that joint counts and assessment of enthesitis were the biggest drivers in treatment decisions. One possible explanation is the inclusion of multiple items in the PsAID questionnaire, only some of which were associated by clinicians with treatment changes (such as the inflamed joint count). Cases where clinicians reported a utility of PsAID-12 scoring in decision-making were related to treatment reduction. With this, PsAID scoring could be used as a tool to increase clinician confidence in treatment de-escalation, it is a quick bedside tool that correlates with multiple measures of disease severity, and generally, patients’ confidence in their interactions and satisfactions with their consultations was high, reflecting a high satisfaction in the physician effort to understand patient concerns. However, those with higher perceived collaboration were more likely to have treatment escalation in mild cases, perhaps reflecting the identification of otherwise undetected symptoms or concerns. Furthermore, it is important to highlight that although most of the researchers in the ASSIST study did not assign an important role to the PsAID scores in the decision to change treatment, there are already studies that demonstrate the predictive capacity of the PsAID in achieving treatment objectives such as the minimal disease activity response [12]. Also, PsAID is able to predict disease flares in recent-onset PsA and as a useful tool in clinical decision-making, including treatment decisions [5].

To date, we are not aware of any research about the treatment decisions made in real-world practice in PsA and how patient and clinician factors influence this. Despite an increasing number of effective therapies and regularly updated evidence-based treatment recommendations, the heterogeneity of the disease means that treatment must be personalized. Composite scores (such as The Psoriatic Arthritis Disease Activity Score (PASDAS)) have previously been shown to have an association with treatment change [12]. However, such scores were not used in this study in order to facilitate assessment of individual aspects of the disease and the relationship of these with treatment change. This study has shown that many different aspects of an individual case are considered within a treatment decision in routine practice.

This study reflects real-world practice with over 500 participants in multiple European countries, to investigate the factors affecting treatment decisions in daily practice. The participants were recruited using systematic sampling with random starting numbers generated for each site to minimize selection bias. The population thus should accurately reflect a real-world clinic population with low levels of average disease activity and treatment escalation in approximately one-third of patients. However, all participants were recruited in specialist PsA clinics so disease activity and treatment decisions may vary in other rheumatology clinics. Furthermore, the clinics used for this study were face-to-face, which may have affected the type of patients in the study. It is likely that results may be different in those with more significant skin disease, although this population does seem to reflect most rheumatology clinic populations.

The enrolment of patients occurred during the years of the COVID-19 pandemic: from July 2021 to March 2022. This potentially had an impact on the patients who were seen in clinic. The pattern of disease seen in clinic could have been different as remote reviews in the pre-COVID-19 era were not as common as in the post-COVID-19 era; however, the impact of this across the included countries is unclear.

Overall, this study highlights the influence of multiple factors on decision-making when reviewing treatments for individuals with PsA. This can help in providing insight into the management of patients with this complex condition.

## Data Availability

Data are available upon reasonable request. Participant-level dataset will be made available upon reasonable request to the Chief Investigator. Some specific data items may not be shared in order to maintain participant anonymity.
